# Clinical effect of high-flow revascularization in microsurgery combined with endoscopic endonasal surgery for skull base tumors with intracranial and extracranial involvement

**DOI:** 10.3389/fsurg.2022.1019400

**Published:** 2023-01-06

**Authors:** Zhi-Qiang Wang, Xiao-Guang Tong

**Affiliations:** ^1^ Clinical College of Neurology, Neurosurgery and Neurorehabilitation, Tianjin Medical University, Tianjin, China; ^2^ Department of Neurosurgery, Tianjin Huanhu Hospital, Tianjin, China

**Keywords:** skull base tumor, high-flow revascularization, internal carotid artery, microsurgery, transnasal endoscopy

## Abstract

**Background:**

The objective of the study is to investigate the surgical methods and clinical effects of high-flow revascularization in microsurgery combined with endoscopic endonasal surgery for skull base tumors with intracranial and extracranial involvement.

**Methods:**

The relationships between skull base tumors and internal carotid artery (ICA), tumor location and size, and the extent of tumor invasion were assessed. Preoperative CT perfusion (CTP), magnetic resonance (MR) perfusion-weighted imaging (PWI) (MR-PWI), and digital subtraction angiography (DSA) were performed to evaluate collateral circulation and brain tissue perfusion. Then craniotomy through the fronto-orbitozygomatic approach was performed, based on which four cases received extended middle skull base approach+Dolenc approach + Fukushima bypass type I, and six cases received extended middle skull base approach+Fukushima bypass type III. After surgery, DSA, CT angiogram (CTA), and CTP/PWI were performed to evaluate the patency of the reconstructed vessels and cerebral perfusion, and contrast-enhanced MRI to evaluate the degree of tumor resection. All patients were followed up for 6–12 months.

**Results:**

Among the 10 cases investigated, gross total resection was achieved in 8 cases, subtotal resection in 1 case, and partial resection in 1 case, as confirmed by CT and enhanced MRI. The patency of revascularization vessels was observed using fluorescein angiography during the operation in all patients and via DSA and CTA postoperatively in nine patients. One patient underwent ventilator-assisted ventilation because of respiratory failure and failed to undergo DSA and CTA. Regarding postoperative complications, one patient developed watershed cerebral infarction on the operated side but no sequelae after drug treatment, three patients developed facial numbness, which improved after 3 months, and two patients experienced worsened diplopia. After 6 to 12 months of follow-up on the nine evaluable patients, the Glasgow Outcome Scale (GOS) was 4–5 after surgery. In addition, 6-month follow-up results showed that one patient with clival chondrosarcoma developed recurrence on contrast-enhanced MRI, while no relapse was observed in the other patients.

**Conclusion:**

For skull base tumors with intracranial and extracranial invasion and involving the ICA, revascularization might improve the total resection rate and reduce the recurrence rate and risk of intraoperative bleeding and postoperative ischemia.

## Introduction

Skull base tumors are deeply located within complex surrounding tissues, and the extracranial part of intracranial and extracranial communicating tumors in the skull base can invade structures such as the sphenoid sinus, maxillary sinus, ethmoid sinus, infratemporal fossa, pterygopalatine fossa, and parapharyngeal space (1), making microscope-assisted surgery for such tumors very challenging (1). Currently, a combination of microscopy and transnasal endoscopy (TNE) has become a hot spot technique in neurosurgery. TNE has the advantage of multiple angles and close observation. Intracranial internal carotid artery (ICA) injury, a serious complication, may easily occur during endoscopic skull base surgery (2, 3), and improper management of the injury may further lead to serious neurological complications and even death (4). Despite various treatments for repairing the injury of ICA, patients are still prone to postoperative occlusion of ICA and postoperative cerebral ischemia, affecting the degree of tumor resection. As a result, these patients are at risk of increased disability and mortality. In recent years, revascularization techniques have been applied in the surgical treatment of skull base tumors involving ICA to prophylactically reduce the risk of intraoperative bleeding and postoperative cerebral ischemia and provide a safeguard for the smooth progress of tumor resection (5).

Intracranial and extracranial communicating tumors may widely invade the skull base structures and involve the ICA and important neural structures, leading to very risky surgery and difficult total resection. Thus, few studies have been conducted on such tumors. This study retrospectively analyzed the clinical data of 10 patients with skull base tumors with intracranial and extracranial invasion and involvement of the ICA who underwent microscopy and TNE. Based on the collected data, we attempted to investigate the surgical methods and clinical efficacy of high-flow revascularization in such patients.

## Materials and methods

### Study subjects

From May 2016 to September 2019, the data of 10 patients with skull base tumors showing intracranial and extracranial invasion and ICA involvement, who underwent surgery assisted by microscopy and TNE at the Tianjin Huanhu Hospital (Tianjin, China), were collected. All patients received head computed tomography (CT), three-dimensional CT reconstruction of vessels, and enhanced magnetic resonance imaging (MRI) to identify the tumor size, location, and extent of invasion. The patients were well-informed about the implications of the treatments, potential risks, and complications and provided signed informed consent. This study has been approved by the Ethics Committee of Tianjin Huanhu Hospital (No. 2021-058).

### Preoperative assessment

Preoperative CT perfusion (CTP), magnetic resonance perfusion-weighted imaging (MR-PWI), and digital subtraction angiography (DSA) were performed to evaluate collateral circulation compensation and brain tissue perfusion. Specifically, perfusion imaging parameters such as mean transit time, time to peak, cerebral blood volume, and cerebral blood flow were recorded. Collateral compensation was graded as follows: Grade I: slow collaterals to the ischemic site, with some defects; Grade II: rapid collaterals to the ischemic site, with some defects; Grade III: collaterals with complete angiographic blood flow of the ischemic site by the late venous phase; grade IV: complete and rapid collateral blood flow to the ischemic site (6). In addition, the balloon occlusion test of ICA was performed to evaluate the compensation of cerebral blood flow and observe the status of the forearm radial artery, and the Allen test to analyze the compensation of the radial artery.

### Surgical procedures

Indications for cerebral revascularization for skull base tumors included (7) the following: (1) benign tumors encasing major blood vessels and with failure to avoid vascular injury during complete resection; (2) malignant tumors involving important blood vessels and with complete resection as the surgical target; (3) major vessels occlusion with cerebral ischemia symptoms or decreased cerebral blood flow reserve; and (4) occurrence of intraoperative major vessel injury that could not be directly repaired.

In addition, two different high-flow revascularization procedures were conducted based on the nature of the tumor, its location, the extent of involvement, and its relationship with the C6 segment of the ICA (ICA-C6).

### Surgical procedures in the Fukushima bypass I group

Extended middle skull base approach + Dolenc approach+Fukushima type I bypass+transnasal endoscopic resection of the extracranial tumor was performed for patients meeting the following criteria: the ICA-C6 segment could be easily exposed early during surgery, and the vessels had suitable conditions to serve as anastomotic ends. The first step was to collect the radial artery group and craniotomy, which were operated on simultaneously. For the collection of the radial artery, preoperative ultrasound was performed to provide accurate location and avoid electrocoagulation of the radial artery. Then a non-absorbable suture was used to ligate the muscular branch vessels of the radial artery, and the radial artery was removed. Papaverine water irrigation was given after removal, and the pressure dilatation technique was used to dilate the removed radial artery to prevent vasospasm. The collected radial artery was wrapped with gauze swabs soaked in normal saline after verifying no leakage and marking the direction of the blood vessel for future use. For craniotomy, the patients were placed in the supine position with the head turned 45° to the unaffected side, the neck flexed 20°, and the top of the head drooped. Such position facilitated the temporal lobe to droop by gravity, thus reducing traction injury. We then performed a skin incision 1 cm below the lower edge of the zygomatic arch, as close as possible to the anterior border of the ear tragus, and went upward along the hairline to reach the midline. For the second step, routine expansion of the middle skull base approach was performed. Separation was performed for the dura mater in the middle skull base, electrocoagulation for the middle meningeal artery at the foramen spinosum, and mobilization or cutting for the greater petrosal nerve. During the process, special care was taken to protect the geniculate ganglion. Third, the bone at the Glasscock's triangle and Kawase's triangle was abraded to fully expose the ICA-C6 segment. The ICA-C6 segment blood flow was blocked with an aneurysm clip, then an end-to-side anastomosis between the proximal end of the radial artery and the ICA-C6 segment, and an end-to-side anastomosis between the distal end radial artery and ICA-C2 segment were performed. After completion of the anastomoses, an aneurysm clip was used to clamp the distal anastomotic end of the ICA-C6 segment and the proximal anastomotic end of the ICA-C2 segment to exclude the ICA between two ends, and intraoperative indocyanine green fluorescein angiography was performed to confirm no blood flow passage in this excluded segment and blood flew in the radial artery graft.

After vascular bypass, the intracranial tumor was removed with special care to protect critical neural structures during tumor resection. If the tumor closely adhered to the excluded ICA segment mentioned above and was challenging to separate, or the malignant tumor invaded the vascular structure, resection of the excluded artery was feasible to achieve complete tumor removal. Careful hemostasis was performed after removing the intracranial tumor and exposing the extracranial tumor. After microscopic complete resection, the skull base dura mater was repaired using autologous fascia lata with watertight closure to prevent cerebrospinal fluid leakage. The epidural autologous fat was used to fill the bone defect, and attention was paid not to form compression on the ICA-C6 segment and reconstructed vessel.

After routine cranial closure, the operating bed was rotated to the position of transnasal endoscopic surgery. The main surgical instrument used was the Karl Storz Endoscopic Sinus Surgery System, a wide angle endoscope with a diameter of 4 mm and length of 18 cm, which provided a field of view at 0°, 30°, and 70°. According to the location of the tumor, the main side of the transnasal endoscopic procedure was selected. The residual tumor was observed closely and exposed with a neuroendoscope at multiple angles, and the optimal angle was determined. Following the surgical concept of Pittsburgh and Kassam, the three-handed technique (three surgeons) or four-handed technique (two surgeons) was used. Through the bilateral nostril approach, a pedicled nasal septum mucosal flap was made to protect the posterior septal artery, following which the lower part of the middle turbinate was removed from the main side without damaging the sphenopalatine artery.

In addition, lateral fracture of the inferior turbinate and removal of the ethmoidal bulla and uncinate process were performed, followed by opening the maxillary sinus, the anterior and posterior ethmoid sinuses, and the sphenoid sinus. Part of the bone of the medial and posterolateral walls of the maxillary sinus was removed to access the pterygopalatine fossa and expose the sphenoid sinus cavity, ethmoid sinus, pterygopalatine fossa, and infratemporal fossa. Afterwards, the tumor was removed as much as possible. If a tumor was present in the sphenoid sinus, intratumoral volume reduction was first performed; the cyst wall of the tumor was freed from the normal bone; and the tumor in this plane was removed. If the tumor had invaded the petroclival region, the transpterygoid approach was selected, followed by bone abrading, after which the surgeons observed for residual tumor. If the lesion had invaded the clivus, the clivus bone was abraded to expose the lesion, and the extracranial tumor was removed. In the case of nasopharyngeal tumors, the tumor was removed deep from the pharyngobasilar fascia, laterally to the parapharyngeal space; and the cartilage of the tubal torus, pharyngeal recess, and Eustachian tube were removed, with attention paid to protect the lower cranial nerves.

After removing the tumor, endoscopic observation was conducted to confirm whether there was leakage after watertight closure of the skull base fascia was performed microscopically. If there was cerebrospinal fluid leakage, skull base reconstruction was performed. Microscopically, artificial dura mater and fascia lata were used to remodel the cavernous sinus. A watertight suture of fascia lata was performed to repair the ruptured dura mater, and autologous dura mater fat was used to fill the petrous bone abrasion site and skull base. Endoscopically, cerebrospinal fluid leakage was examined through the nose, and “sandwich” skull base reconstruction was given in combination with skull base repair to maximize the prevention of cerebrospinal fluid leakage.

### Surgical procedures in Fukushima bypass III group

Extended middle skull base approach for resection of intracranial tumor was selected with Fukushima bypass III and transnasal endoscopic resection of the extracranial tumor for patients meeting the following criteria: ICA-C6 segment was not suitable for exposure or seriously involved by tumors, or the vascular lumen was narrow, causing poor blood supply and the compensation of contralateral part of anterior and posterior communicating arteries. The patient's position and the head surgical incision were the same as before. In this group of patients, an additional “S”-shaped incision (about 10 cm in length) was made, starting with the plane of carotid bifurcation as the center and moving along the anterior border of the sternocleidomastoid muscle.

The procedures of radial artery collection and craniotomy were performed simultaneously. The team that operated the radial artery acquisition and treatment also operated the Fukushima bypass I group. At the same time, the team for craniotomy first completed the procedures on the neck. Specifically, the surgeons slightly lifted the mandible angle of patients and exposed the anterior triangle of the neck as far as possible to facilitate the exposure of the cervical vessels (sometimes requiring anesthesia cooperation). The operation was performed from the lower to the upper region to fully expose the cervical vessels. The external carotid artery was treated and served as the origin of the Fukushima III end-to-end anastomosis. Then the incision for craniotomy was made about 5-cm anterior to the ear tragus, and after the subcutaneous layer was freed, a guide tube of approximately 1-cm diameter was passed through the position close to the ear tragus to reach the neck incision. The tube was inserted at the beginning of the surgery, which was beneficial for subcutaneous dilatation. The posterior part of the zygomatic root was ground slightly flat to prevent compression on the radial artery graft and avoid an effect on the patency of the blood flow. During the tube insertion procedures, careful attention was paid to protect the neighboring nerves: first, the guide tube was as close as possible to the ear tragus, and if it was close to the midpoint of the zygoma, the insertion could damage the nerve branches. Second, the subcutaneous tunnel was short, and the surgeons tried to insert the tube below the tragus and then backward into the neck incision, thus reducing compression. The neck incision was covered with gauze swabs soaked in normal saline for future use.

Afterwards, the procedures for routine expansion of the middle skull base approach were the same as the Fukushima bypass I group, but without exposing the ICA-C6 and ICA-C2 segments. End-to-end anastomosis between the proximal end of the radial artery and external carotid artery was performed, following which the absence of any leakage at the anastomosis site was confirmed by the test of flow intensity in the intracranial radial artery, and the graft passed flow pressure and patency were measured. If the flow was poor, the reasons were required to be analyzed, such as anastomotic stenosis, radial artery spasm, and subcutaneous tunnel compression. With satisfactory blood flow pressure and patency of reconstructed vessels, the surgeons anastomosed the distal end of the radial artery to the intracranial vessels, with intracranial M2 superior or inferior trunk (end of M1 segment could be selected if necessary) as the recipient vessel. The end-to-side anastomosis was mostly selected. Upon completion of the anastomosis, intraoperative indocyanine green fluorescein angiography was performed to confirm the patency of blood flow. Then the proximal ophthalmic artery segment of the ICA was clamped with aneurysm clips, and the cervical segment of the ICA was ligated with non-absorbable sutures. After that, the surgeons determined no blood flow in the excluded ICA, and subsequently, the intracranial tumor was carefully removed. Following this, the TNE-assisted operation was performed as before.

It was recommended to reexamine DSA immediately after the operation to confirm whether the blood flow was adequate. Following satisfactory blood flow and full recovery after anesthesia, the patients were taken to the intensive care unit. If bypass flow was difficult to fill the affected hemisphere, it was necessary to rule out the causes, namely, muscle, bone, skin compression, radial artery spasm, and inadequate pressure at the external carotid artery anastomosis site. When necessary, the indicated location was opened again for repair. It was best to first incise the neck and longitudinally cut the revascularized radial artery to determine which end had problems with flow, following which the incision of the radial artery was sutured after the surgeons confirmed the proximal and distal pressures of the revascularized radial artery were sufficient, and the blood flow was unobstructed. In the case of severe spasms, balloon dilatation could be used. Then the surgery team of the Endoscopic Skull Base Center performed the extracranial tumor resection described before.

### Perioperative management

Before surgery, relevant evaluation was completed, and patients received oral aspirin (100 mg/time, once daily) or clopidogrel (for patients with aspirin resistance by thromboelastography; 75 mg/time, once daily) for at least 1 week. After surgery, the patients were given aspirin (100 mg/time, once daily) or clopidogrel (for patients with aspirin resistance by thromboelastography; 75 mg/time, once daily) for at least 1 year. They were monitored for the following: swelling and errhysis in the neck, drainage volume of intracranial drainage tube and shape of drainage content, blood pressure (required range: systolic blood pressure of upper limb cuff between 120 mmHg and 140 mmHg, or systolic blood pressure of lower limb between 100 mmHg and 120 mmHg if the upper limb blood pressure was difficult to measure). Patients were reexamined with brain CT the next day after operation, and the nasal packing material was removed 2–3 days after the operation. On postoperative day 3, brain CT, CT angiogram (CTA), DSA, CTP, and MRI were reexamined according to the patients' condition. On postoperative day 30, the patients underwent outpatient reexamination at the Endoscopic Skull Base Center to remove scabs in the nasal cavity and observe whether there were adhesions, and their endocrine parameters were assessed. At 3 months after surgery, the patients were reexamined in the ophthalmology, otorhinolaryngology, and neurosurgery clinics. At 6 to 12 months after surgery, DSA or CTA was repeated to assess the patency of the anastomotic vessels and to assess the presence or absence of cerebral hypoperfusion. From 3 months after surgery, plain and enhanced MRI were performed regularly to evaluate tumor recurrence.

### Postoperative follow-up

Intraoperative fluorescein angiography and postoperative DSA or CTA were performed to assess the patency of the anastomotic vessels, and CTP was reexamined to evaluate brain tissue perfusion. No residual tumor tissue was observed under fiberscope and TNE during operation, and plain and enhanced MRI were performed after operation to evaluate the degree of tumor resection: gross total resection: no residual tumor; subtotal resection: tumor resection of 80% and more; and partial resection: tumor resection of less than 80%. Postoperative neurological recovery and quality of life were estimated using the Glasgow Outcome Scale (GOS) (8), which is an objective assessment to assess patients' degree of recovery, and was graded as follows: Scale 1, death; Scale 2, vegetative state with only minimal response (e.g., periods of spontaneous eye-opening); Scale 3, severe disability, unable to live independently and requiring care in daily life; Scale 4, moderate disability, capable of living independently and working under protection; Scale 5, good recovery, capable of returning to everyday life but with mild deficit.

## Results

### Baseline data of patients

This study included ten patients, two males and eight females, with intracranial and extracranial communicating skull base tumors involving the ICA (Table 1). They were aged 31–68 years, with a median age of 49 years. Five of the 10 patients had skull base meningiomas involving the C6 to C2 segments of the ICA, sphenoid ridge, temporal lobe, cavernous sinus, ethmoid sinus, sphenoid sinus, infratemporal fossa, pterygopalatine fossa, petrous bone, and petrous apex. Three of the 10 patients had refractory pituitary adenomas involving the C6 to C2 segments of the ICA, parasellar region, optic chiasma, third ventricle, cavernous sinus, ethmoid sinus, sphenoid sinus, pterygopalatine fossa, and infratemporal fossa. One patient had clival chondroma involving the C6–C3 segment of the ICA, petrous bone, basilar venous plexus, cavernous sinus, posterior clinoid process, and Meckel's cave. One patient had recurrent fibrous dysplasia combined with internal carotid artery aneurysm. After embolization of the bilateral cavernous internal carotid artery aneurysms, the aneurysm cavity in the cavernous sinus segment of the left internal carotid artery remained, and the tumor lesion that protruded into the pterygoid sinus, posterior ethmoid sinus, and the base of the anterior cranial fossa was damaged.

**Table 1:** Basic clinical information of the patients.

Patients	Age	Gender	Tumor type	Preoperative-BOT	ICA involvement	Type of surgery/bypass	Outcome
No. 1	64	Male	Meningiomas	Left side ACA advantage	Right ICA end compression thinning, right MCA proximal occlusion	STA-M3	–
No. 2	68	Female	Meningiomas	BOT+	Right side C4 wrapping	Double STA-MCA combined with C4 end-to-end anastomosis	Symptoms improved after one month
No. 3	56	Female	Meningiomas	Left side ACA advantage	Right C4 encapsulation, ICA end and MCA occlusion	STA-M2	Dead
No. 4	31	Female	Refractory pituitary adenomas	—	Arterial injury after right C4 pituitary tumor surgery	C2-RA-M2 Fukushima Bypass	—
No. 5	48	Female	Refractory pituitary adenomas	ACA partial development, MCA no development	Left C3 and C4 encapsulation, C4 hemorrhage pseudoaneurysm formation	C2-RA-C6	—
No. 6	54	Female	Clival chondroma	BOT−	Left C2-4 circumferential stenosis	C2-RA-C6	Endoscopic total resection after 5 months of recurrence
No. 7	45	Male	recurrent fibrous dysplasia combined with internal carotid artery aneurysm	—	—	ECA-RA-M2 Bypass	—
No. 8	60	Female	Refractory pituitary adenomas	ACA partial development, MCA no development	Right C2-C6 encapsulated; left C2-C6, BA and double A1 displaced by compression	ECA-RA-M2	Obstructive hydrocephalus not resolved, discharged with tube
No. 9	51	Female	Meningiomas	BOT−	Right C2 partially involved, C4 encapsulated	C2 end-to-end anastomosis	—
No. 10	56	Female	Meningiomas	Left side ACA advantage	C4 wrap around, C5 squeeze shift	Direct anastomosis	—

ICA, internal carotid artery; MCA (M2, M3), middle cerebral artery; BOT, ballon occlusion test; ACA, anterior cerebral artery; STA, superficial temporal artery.

The main clinical symptoms were as follows: decreased visual acuity in one case, visual loss in one case, facial hypoesthesia in five cases, diplopia in three cases, inability of eyeball abduction in three cases, epistaxis in two cases, swallowing dysfunction in one case, and headache with dizziness in one case. Regarding collateral circulation, three patients had compensation of the anterior communicating artery, two patients had compensation of the posterior communicating artery, and five patients had no compensation of the anterior and posterior communicating arteries.

Four patients underwent Fukushima high-flow bypass type I, and six received Fukushima high-flow bypass type III according to the relationship between the tumor and the ICA and the status of compensation of collateral circulation.

### Evaluation of revascularization and tumor resection

**Figure 1:**
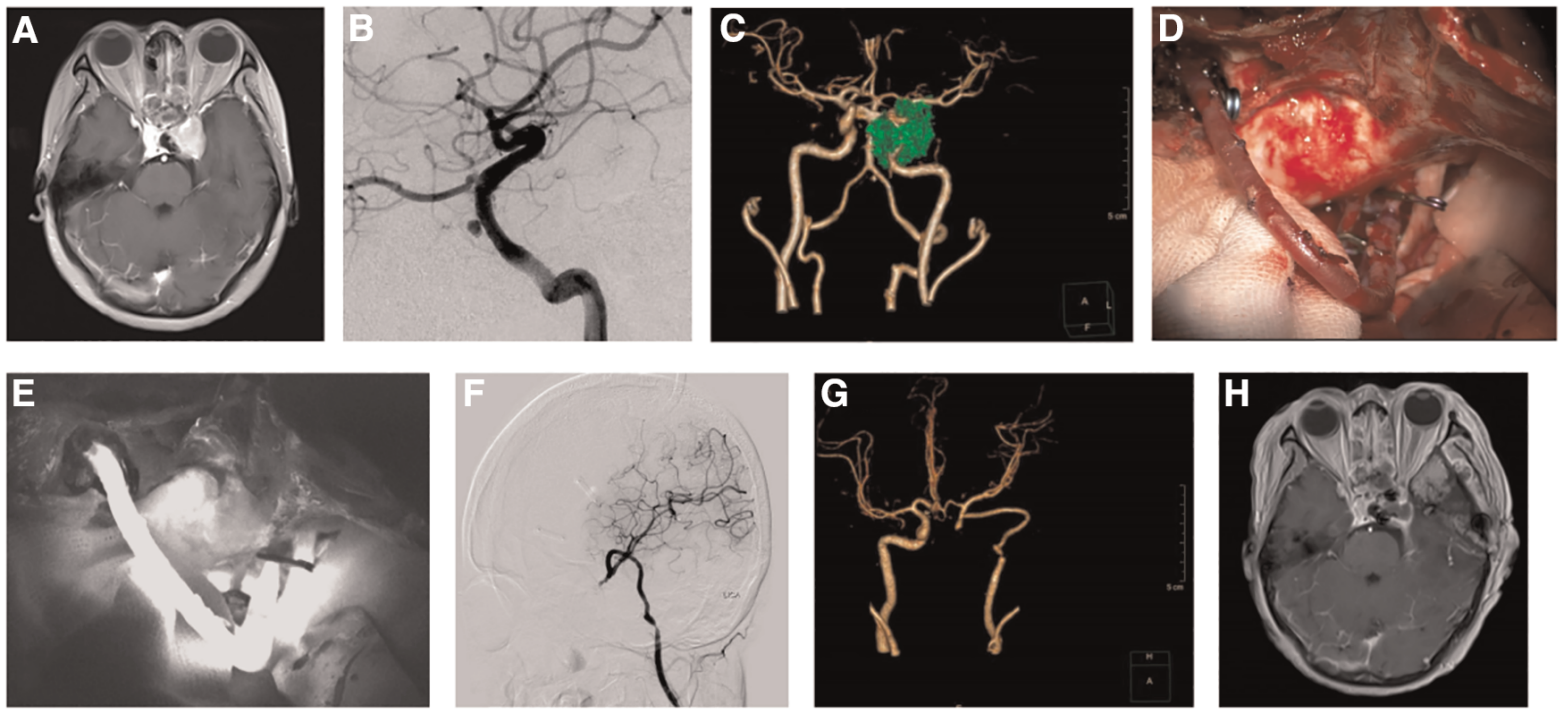
Images of a patient before and after Fukushima bypass type I. (**A**) Enhanced magnetic resonance imaging (MRI) showing a pituitary adenoma in the sellar region involving the cavernous sinus and encasing the left internal carotid artery. (**B**) Digital subtraction angiography (DSA) showed pseudoaneurysm of the internal carotid artery. (**C**) Computed tomography angiogram (CTA) showing tumor encasing the left internal carotid artery and aneurysm. **(D**). Intraoperative image of Fukushima bypass. (**E**) Intraoperative fluorescein angiography showing patent bypass blood flow. (**F**) Postoperative reexamination of MRI showing subtotal resection of the tumor. (**G**) Postoperative CTA showing patent blood flow in radial artery graft. (**H**) Postoperative DSA showing patent blood flow after vascular anastomosis.

**Figure 2:**
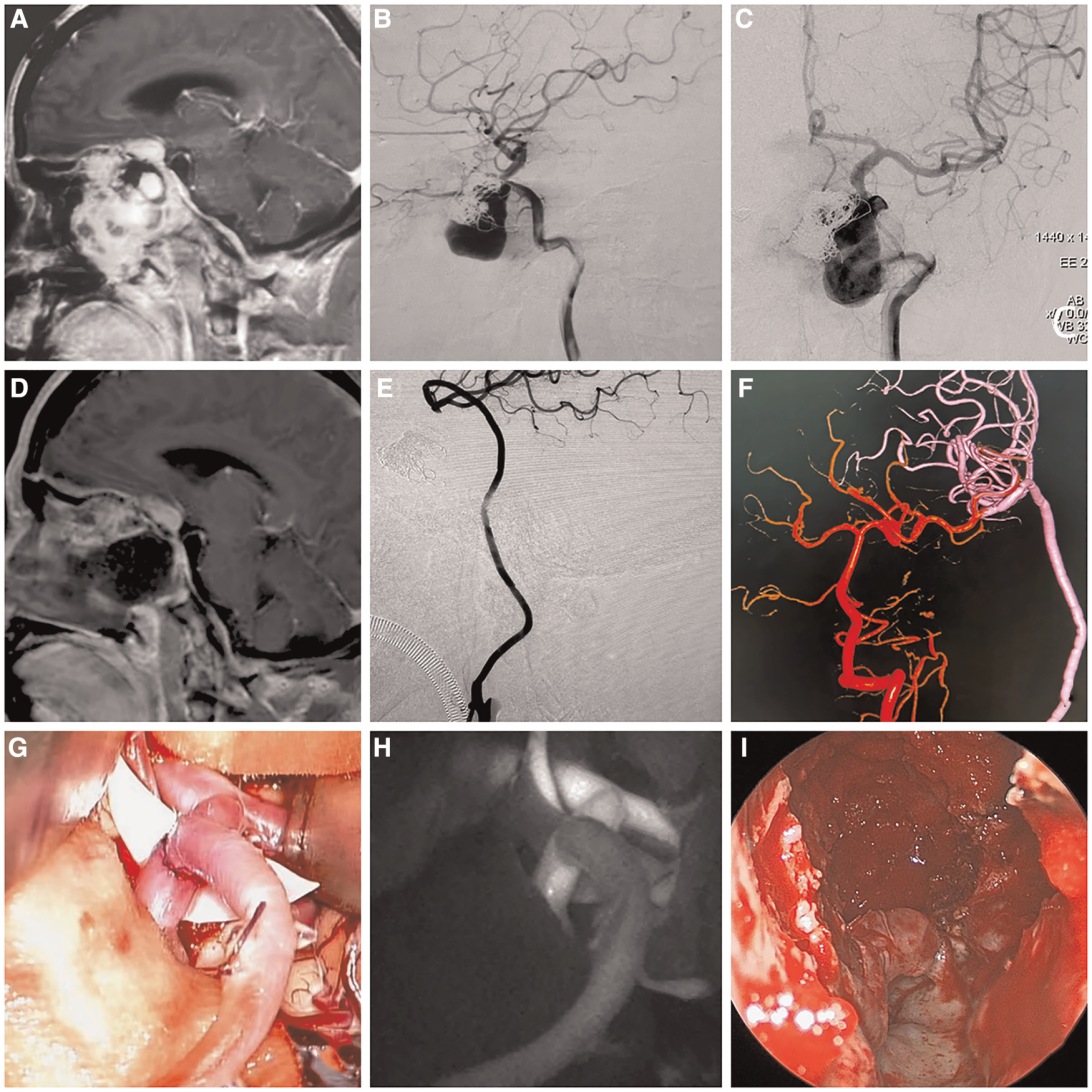
Images of a patient with recurrent fibrous dysplasia combined with internal carotid artery aneurysm who received Fukushima bypass type III (ECA-RA-M2) combined with transnasal endoscopic surgery to remove the affected bone, achieved optic nerve decompression and resection of the left internal carotid artery aneurysm. (**A**) Preoperative enhanced cranial magnetic resonance imaging (MRI) showing the internal carotid artery was invaded, and an aneurysm was formed. (**B, C**) Preoperative lateral and front digital subtraction angiography (DSA) projections showing the internal carotid artery and aneurysm. (**D**) Postoperative enhanced cranial MRI showing subtotal resection of the tumor and adequate decompression. (**E, F**) Postoperative cerebral DSA showing good patency of the radial artery graft, no visualization of ICA-C1 to ICA-C4 segments, and anterior cerebral circulation blood supply from posterior cerebral circulation. (**G**) Intraoperative image of Fukushima bypass type III (ECA-RA-M2). (**H**) Intraoperative fluorescein angiography showing good patency of the radial artery graft. (**I**) Intraoperative images of endoscopic subtotal resection of tumors.

In this study, intraoperative fluorescein angiography showed good patency of the radial artery graft in 10 patients, and postoperative CTA and DSA also revealed good patency of the reconstructed vessels and no blood flow in the excluded vessels. In addition, CTP showed no cerebral hypoperfusion on the reconstructed side after surgery. In terms of tumor resection, contrast-enhanced MRI of the brain confirmed total resection in eight cases, subtotal resection in one case, and partial resection in one case. Figures 1 and 2 are typical preoperative and postoperative images of a patient who underwent Fukushima bypass type I and a patient who underwent Fukushima bypass type III, respectively.

### Complications

One patient underwent ventilator-assisted ventilation due to respiratory failure and failed to receive DSA and CTA. Postoperatively, one patient developed watershed cerebral infarction on the operated side, but no sequelae after antiplatelet and anticoagulation therapy. Three patients presented with facial numbness, which improved after 3 months. Two patients developed diplopia.

### Follow-up and prognosis

Nine patients were followed up for 6 to 12 months. The last follow-up showed that none of the nine patients had new-onset cerebral ischemia or neurological dysfunction, and seven of nine patients underwent DSA reexamination, with six showing patency of the reconstructed vessel and one having mild stenosis of the reconstructed vessel. All nine patients underwent CTP examination, which revealed no cerebral hypoperfusion on the reconstructed vessel side. One patient developed postoperative cerebral infarction, one patient with clival chondrosarcoma developed a recurrence, which was identified on a contrast-enhanced MRI 6 months after surgery, and the remaining patients had no recurrence. During the follow-up period, two cases of diplopia were improved but not cured at 12 months after surgery. Patients with facial numbness had poor recovery. After 6 to 12 months of follow-up, the GOS of nine patients reached scale 4–5, indicating that they needed no assistance in everyday life with minor neurological and psychological deficits.

## Discussion

Skull base tumors with intracranial and extracranial involvement account for about 10% of all skull base tumors. They are defined as tumors originating from the intracranial or extracranial region but passing through the skull base bone and dura mater to cause intracranial and extracranial communication (9). For patients with intracranial invasion, the tumor widely involves the skull base bone, dura mater, orbital periosteum, and other structures; and surgical resection and radiotherapy are limited by anatomical factors. As a result, such patients have poor overall efficacy and are prone to recurrence, attributed to residual tumor tissues (10).

Domestic and foreign literature reported that the perioperative mortality of skull base tumors had decreased significantly to only 0%–4.7%, but there are significant individual differences in the total resection rate (62.9%–100%) and the incidence rate of postoperative complications (20%–50%) (5, 6, 11). Microscope-guided craniotomy and TNE surgery complement each other and are an important combination for treating intracranial and extracranial communicating skull base tumors because together they can provide a good visual field and operating space. However, damage to the ICA may easily occur during TNE-assisted procedures because of the absence of bone around the ICA, abnormal course of the ICA, thin ICA wall, encasement or displacement of the ICA, history of secondary surgery or radiotherapy, and lack of appropriate instruments for transnasal endoscopic skull base surgery. Thus, acquisition of expertise in endonasal surgery, engagement in dedicated training programs, ongoing and intensive learning (i.e., mastering the perfect knowledge of nasal cavities and anatomy of the sellar region learned from dissection practice in the laboratory, endoscopic manipulation techniques, understanding of preoperative imaging, etc.), careful patient selection and multidisciplinary teamwork are key to achieve satisfactory surgical outcomes.

Patients with a high risk of intraoperative ICA or even requiring the sacrifice of the ICA might benefit from balloon occlusion testing preoperatively (2), which was shown to be the most effective method to assess collateral circulation (12). Although theoretically, arterial sacrifice is possible in patients obtaining a negative result from the testing, 5%–20% of the patients will clinically experience cerebral ischemia after surgery (13, 14). Lawton et al. recommended vascular graft bypass surgery in all patients who require occlusion of the main intracranial arteries (15). After revascularization, vascular grafts provide adequate blood supply to the brain (16). Relevant studies have shown that patients with skull base tumors treated using combined vascular reconstruction had higher total resection rate and longer disease-free survival, and their incidence of perioperative cerebral ischemia was also significantly decreased (6, 13, 14, 17). In this study, according to the location and nature of the tumor and the relationship between the tumor and the ICA, a high-flow bypass was selected, and tumor resection was performed after the surgeons confirmed the reconstructed vessels were unobstructed and the corresponding segment of the ICA was ligated. The follow-up results revealed that gross total resection of the tumor was achieved in eight cases, subtotal resection in one case, and partial resection in one case. Nine patients were followed up for 6 to 12 months, of whom one patient with clival chondrosarcoma showed recurrence on contrast-enhanced MRI at 6-month follow-up, and all nine patients underwent CTP examination suggesting no cerebral hypoperfusion on the reconstructed vessel side. These results are generally consistent with previous studies. In addition, our postoperative follow-up found that one patient developed watershed cerebral infarction on the operated side but no sequelae after drug treatment; three patients developed facial numbness, which was improved after 3 months; two patients developed diplopia; and one patient developed respiratory failure. The prognosis of nine patients who were followed up postoperatively was assessed using the GOS, and all of them were found to have a GOS score of 4–5. These results suggest that high-flow revascularization could reduce the incidence of postoperative complications associated with skull base tumor surgery.

This study had some limitations. First, the sample size was small and lacked relevant controlled analysis for clear statistical inference. Therefore, a large sample of prospective controlled studies is needed to further verify the conclusions of this study. Second, the follow-up time was not long enough, and we did not assess the long-term efficacy and complications. Third, we investigated patients with skull base tumors showing intracranial and extracranial invasion and ICA involvement, irrespective of the type and stage of the tumor and despite promising outcomes observed in most patients, the treatment approach was quite aggressive, and indications for surgery should be improved. In their study, Kalani et al. performed maximal surgical intervention in 18 patients with tumors involving the ICA at the skull base and found that the patients' survival was dismal and the rate of complication was high despite ICA sacrifice at the skull base with revascularization (18). Thus, considering this was our preliminary study investigating the potential efficacy of high-flow revascularization in microsurgery combined with endoscopic endonasal surgery, the indications for such surgery should be further clarified, with careful evaluation of the risk-to-benefit ratio, in future studies.

## Conclusion

For complex intracranial and extracranial communicating tumors widely involving the sellar region, clivus, and petrous apex region of the skull base, a combination of the fronto-orbitozygomatic approach using a microscope and endoscopic transnasal approach may improve the curative rate and could reduce postoperative complications. High-flow revascularization technique and exclusion of the ICA involved by the tumor may decrease the risk of intraoperative bleeding, improve the gross total resection rate, and reduce damage to critical cranial nerves. Collectively, the findings from this study provide additional insight into the gross total resection rate of skull base tumors with intracranial and extracranial involvement, which was shown to reduce the recurrence rate and the risk of intraoperative bleeding and postoperative ischemia. However, it should be noted that the results presented here were from a retrospective observational study and the treatments performed in these settings need further investigation. A larger cohort, prospective settings, well-designed additional group comparison and longer follow-ups are needed to validate these findings.

## Data Availability

The original contributions presented in the study are included in the article/Supplementary Material, further inquiries can be directed to the corresponding author/s.
